# ABO Blood Group, SARS-CoV-2 Infection, and Risk of Venous Thromboembolism: Population-Based Cohort Study

**DOI:** 10.1177/10760296211008986

**Published:** 2021-04-08

**Authors:** Joel G. Ray, Marian J. Vermeulen, Michael J. Schull, Alison L. Park

**Affiliations:** 1 Department of Medicine, St. Michael’s Hospital, 7938 University of Toronto, Toronto, Ontario, Canada; 2 Department of Obstetrics and Gynaecology, St. Michael’s Hospital, 7938 University of Toronto, Toronto, Ontario, Canada; 3 50010 ICES, Toronto, Ontario, Canada; 4 7938 University of Toronto, Toronto, Ontario, Canada; 5 Sunnybrook Research Institute, 7938 University of Toronto, Toronto, Ontario, Canada

**Keywords:** non-O blood, blood group, SARS-CoV-2, COVID-19, pulmonary embolism, deep vein thrombosis, venous thromboembolism

Persons with non-O blood group (i.e., A, AB or B) may be at higher risk of venous thromboembolism (VTE) than those with O blood (odds ratio 1.79, 95% CI 1.56 to 2.05).^1^ Non-O blood groups may also be predisposed to severe acute respiratory syndrome coronavirus 2 (SARS-CoV-2).^2^ During the recent SARS-CoV-2 pandemic, a high rate of VTE was observed among infected patients.^3^ Lung tissue specimens from coronavirus disease 2019 (COVID-19) patients showed a preponderance of alveolar capillary microthrombi.^4^ A unique COVID-19-associated predisposing coagulopathy (CAC) was proposed.^5^ It is not known whether SARS-CoV-2 infected patients with non-O blood groups are especially at higher risk of VTE.

This population-based retrospective cohort study was performed across Ontario, Canada, where health care is universal. Patient-level datasets capturing all inpatients, emergency department and outpatient visits, medical imaging billings, and SARS-CoV-2 laboratory tests were linked using unique encoded identifiers and analyzed at ICES (Table S1). The determination of pulmonary embolism (PE) or deep vein thrombosis (DVT) required a coded diagnosis of either condition, in conjunction with performance of objective imaging (Table S1).

Included were individuals aged 16+ years, who had undergone ABO determination between January 2007 and December 2019, and who then underwent SARS-CoV-2 viral RNA PCR testing between January 15, 2020 and June 30, 2020.

First, the overall risk of PE or DVT was determined in relation to non-O vs. O blood groups. Next, PE or DVT risk was assessed in association with SARS-CoV-2 positivity vs. negativity. Third, the influence of O blood group was stratified by those with a positive or negative SARS-CoV-2 test.

Cox proportional hazard models generated incidence rates and hazard ratios (HR) and 95% CI, with time zero (t_0_) first set to the date of ABO testing, and then re-set to the date of SARS-CoV-2 specimen collection. Censoring, starting on the date of SARS-CoV-2 testing, was on death or arrival to the end of the study period of July 31, 2020. HR were adjusted for demographics and comorbidities.

In total, 222,670 individuals were included in the final cohort. Their mean age was 51 years at ABO testing and 54 years at SARS-CoV-2 testing; 71% were female (Table 1). No important differences were seen in the rate of prior VTE, or for any other comorbidities (Table 1).

**Table 1.** Characteristics of 222,670 Individuals in Ontario, Canada, With Known ABO Blood Group, and Who Subsequently Underwent SARS-CoV-2 Viral RNA PCR Testing Between January 15, 2020, and June 30, 2020.

Characteristic		O blood group (N = 98,664)	Non-O blood groups* (N = 124,006)	Standardized difference
At the ABO specimen date (first time zero)				
Mean (SD) age, y		50.7 (21.3)	50.7 (21.3)	.00
At the SARS-CoV-2 specimen date (second time zero)				
Mean (SD) age, y		54.4 (20.8)	54.2 (20.7)	.01
Female		70,485 (71.4)	87,986 (71.0)	.01
Income quintile (Q)	Q1 (lowest)	21,113 (21.4)	25,558 (20.6)	.02
	Q2	19,063 (19.3)	23,703 (19.1)	.01
	Q3	17,836 (18.1)	22,649 (18.3)	.00
	Q4	17,131 (17.4)	21,487 (17.3)	.00
	Q5 (highest)	15,738 (16.0)	20,169 (16.3)	.01
Within 5 years before the SARS-CoV-2 specimen collection date				
Pulmonary embolism or deep vein thrombosis		3,793 (3.8)	5,543 (4.5)	.03
Malignancy		28,230 (28.6)	35,481 (28.6)	.00
Cardiac ischemia/arrhythmia		14,382 (14.6)	18,292 (14.8)	.00
Chronic kidney disease		11,446 (11.6)	14,088 (11.4)	.01
Stroke or transient ischemic attack		4,013 (4.1)	4,966 (4.0)	.00
Anemia		21,489 (21.8)	26,856 (21.7)	.00
Any time before the SARS-CoV-2 specimen collection date				
Congestive heart failure		10,834 (11.0)	13,859 (11.2)	.01
Diabetes mellitus		21,057 (21.3)	27,147 (21.9)	.01
Chronic hypertension		41,109 (41.7)	50,612 (40.8)	.02
Asthma		20,020 (20.3)	24,855 (20.0)	.01
COPD		15,885 (16.1)	19,642 (15.9)	.01
Dementia or frailty		37,176 (37.7)	45,891 (37.0)	.01
HIV or organ transplant		914 (0.9)	1,188 (1.0)	.00
Median (IQR) duration of follow-up from the ABO specimen date, y		3.1 (1.6-5.4)	3.1 (1.6-5.4)	.00
Median (IQR) duration of follow-up from the SARS-CoV-2 specimen date, y		0.2 (0.1-0.3)	0.2 (0.1-0.3)	.00

All data are presented as a number (%) unless otherwise indicated.

*Non-O blood groups are A, AB and B.

Starting t_0_ from ABO testing, the aHR was higher for PE (1.14, 1.06 to 1.23) and DVT (1.12, 1.05 to 1.19) comparing O vs. non-O blood groups (Table 2).

**Table 2.** Non-O Blood Groups, SARS-CoV-2 Infection, and Their Combination, and Future Risk of Pulmonary Embolism or Deep Vein Thrombosis.

	Outcome	Exposure	No. (incidence rate per 1000 person-years, 95% CI)	Hazard ratio for VTE (95% CI)	
				Unadjusted	Adjusted
All patients: t_0_ set at ABO test date (N = 222,670)	Pulmonary embolism	O blood (N = 98,664)	1213 (3.2, 3.0 to 3.4)	1.00 (Referent)	1.00 (Referent)
		Non-O blood (N = 124,006)	1774 (3.7, 3.5 to 3.9)	1.16 (1.08 to 1.25)	1.14 (1.06 to 1.23)*
	Deep vein thrombosis	O blood (N = 98,664)	1814 (4.8, 4.6 to 5.0)	1.00 (Referent)	1.00 (Referent)
		Non-O blood (N = 124,006)	2605 (5.5, 5.2 to 5.7)	1.14 (1.07 to 1.21)	1.12 (1.05 to 1.19)*
All patients: t_0_ set at SARS-CoV-2 test date (N = 222,670)	Pulmonary embolism	SARS-CoV-2 -VE (N = 215,679)	633 (14.8, 13.6 to 15.9)	1.00 (Referent)	1.00 (Referent)
		SARS-CoV-2 +VE (N = 6991)	33 (21.3, 14.1 to 28.6)	1.55 (1.10 to 2.21)	1.64 (1.15 to 2.33)^†^
	Deep vein thrombosis	SARS-CoV-2 -VE (N = 215,679)	658 (15.3, 14.2 to 16.5)	1.00 (Referent)	1.00 (Referent)
		SARS-CoV-2 +VE (N = 6991)	16 (10.3, 5.3 to 15.4)	0.71 (0.43 to 1.16)	0.74 (0.45 to 1.22)^†^
SARS-CoV-2 ---VE: t_0_ set at SARS-CoV-2 test date (N = 215,679)	Pulmonary embolism	O blood (N = 95,813)	234 (12.3, 10.7 to 13.8)	1.00 (Referent)	1.00 (Referent)
		Non-O blood (N = 119,866)	399 (16.8, 15.1 to 18.4)	1.36 (1.16 to 1.60)	1.33 (1.13 to 1.56)^†^
	Deep vein thrombosis	O blood (N = 95,813)	290 (15.2, 13.5 to 17.0)	1.00 (Referent)	1.00 (Referent)
		Non-O blood (N = 119,866)	368 (15.5, 13.9 to 17.0)	1.02 (0.87 to 1.18)	0.98 (0.84 to 1.14)^†^
SARS-CoV-2 +VE: t_0_ set at SARS-CoV-2 test date (N = 6991)	Pulmonary embolism	O blood (N = 2851)	12 (19.2, 8.3 to 30.1)	1.00 (Referent)	1.00 (Referent)
		Non-O blood (N = 4140)	21 (22.8, 13.0 to 32.6)	1.20 (0.59 to 2.43)	1.14 (0.56 to 2.31)^†^
	Deep vein thrombosis	O blood (N = 2851)	9 (14.4, 5.0 to 23.8)	1.00 (Referent)	1.00 (Referent)
		Non-O blood (N = 4140)	7 (7.6, 2.0 to 13.2)	0.53 (0.20 to 1.42)	0.48 (0.18 to 1.30)^†^

Abbreviations: t_0_, time zero for starting follow-up; VTE, venous thromboembolism; -VE, negative SARS-CoV-2 test; +VE, positive SARS-CoV-2 test.

Shown are results for all 222,670 patients, with time zero (t_0_) first set to the date of ABO testing (top blue), and then t_0_ re-set to the date of SARS-CoV-2 testing, presented by SARS-CoV-2 infection (upper middle red), and then by non-O vs. O blood groups further stratified by SARS-CoV-2 negative (lower middle maroon) or SARS-CoV-2 positive (lower green) status.

*Adjusted for age and sex at the ABO specimen date, and a history of venous thromboembolism, malignancy, cardiac ischemia or arrhythmia, or chronic kidney disease diagnosed – each within 5 years before the ABO specimen collection date; as well as a history of diabetes mellitus or congestive heart failure – each diagnosed any time before the ABO specimen collection date. Note: In this model, censoring on death began on the date that the patient underwent SARS-CoV-2 testing.

^†^ Adjusted for age and sex at the SARS-CoV-2 specimen date, and a history of venous thromboembolism, malignancy, cardiac ischemia or arrhythmia, or chronic kidney disease diagnosed – each within 5 years before the SARS-CoV-2 specimen collection date; as well as a history of diabetes mellitus or congestive heart failure – each diagnosed any time before the SARS-CoV-2 specimen collection date.

Starting t_0_ from SARS-CoV-2 testing, the overall incidence rate of VTE rose considerably. Those with vs. without SARS-CoV-2 had a higher aHR of PE (1.64, 1.15 to 2.33), but not DVT (0.74, 0.45 to 1.22) (Table 2). The aHR for PE was higher among non-O blood groups in patients without SARS-CoV-2 infection (1.33, 1.13 to 1.56), but not those with SARS-CoV-2 infection (1.14, 95% CI 0.56 to 2.31) (Table 2).

These findings confirm a higher risk of PE and DVT with non-O blood groups.^1^ The risk of PE, but not DVT, was elevated in those with SARS-CoV-2, as also suggested elsewhere, possibly due to pulmonary immunothrombi arising in situ.^3,4^ Preliminarily, non-O blood group did not appreciably modify the risk of VTE in SARS-CoV-2 positive patients, yet, the number of VTE events was low, producing imprecise risk estimates.

While a coded diagnosis and objective imaging for VTE were required, imaging tests and their reports were not available. This study also did not quantify illness severity among SARS-CoV-2 infected patients, or their receipt of anticoagulants. Those most profoundly affected by the infection may have died prior to SARS-CoV-2 testing, such as from a fatal PE or respiratory failure, so the influence of ABO status among that group could not be determined herein.

Those with O blood group express lower plasma levels of factor VIII and von Willebrand factor, which may protect them against VTE.^1^ SARS-CoV-2 infected patients exhibit much higher levels of both of these factors,^5^ potentially blunting any protective effect from O blood group. The interaction between these and other blood factors and ABO status in patients with SARS-CoV-2 infection should be properly studied.

## Supplemental Material

Supplemental Material, sj-pdf-1-cat-10.1177_10760296211008986 - ABO Blood Group, SARS-CoV-2 Infection, and Risk of Venous Thromboembolism: Population-Based Cohort Study Click here for additional data file. Supplemental Material, sj-pdf-1-cat-10.1177_10760296211008986 for ABO Blood Group, SARS-CoV-2 Infection, and Risk of Venous Thromboembolism: Population-Based Cohort Study by Joel G. Ray, Marian J. Vermeulen, Michael J. Schull and Alison L. Park in Clinical and Applied Thrombosis/Hemostasis
