# Supplementary material for: ABO Blood Group, SARS-CoV-2 Infection, and Risk of Venous Thromboembolism: Population-Based Cohort Study
Source: Clin Appl Thromb Hemost. 2021 Apr 8;27:10760296211008986. doi: 10.1177/10760296211008986 (PMC8040562; doi:10.1177/10760296211008986)
Supplement: Supplemental Material, sj-pdf-1-cat-10.1177_10760296211008986 - ABO Blood Group, SARS-CoV-2 Infection, and Risk of Venous Thromboembolism: Population-Based Cohort Study [file sj-pdf-1-cat-10.1177_10760296211008986.pdf]

Table S1. Variables used to define cohort entry and exclusion criteria, as well as study exposures, outcomes and adjustment.

Assessment	Timing	Disease, procedure or condition	CIHI-DAD, SDS or NACRS ICD-10-CA diagnosis or CCI procedure codes	OHIP ICD-9 diagnosis or fee codes; or other data source	Validation studies or documentation for some codes
Inclusion criteria	ABO-Rh specimen date January 2007 to December 2019	Individuals with an ABO-Rh blood group test result in Ontario, Canada	--	LOINC codes 882-1, 883-9, 10331-7 in the Ontario Laboratory Information System (OLIS) - includes most outpatient laboratory information in Ontario	--
Exclusion criteria	SARS-CoV-2 specimen date January 15, 2020 to June 30, 2020	Individuals without a SARS-CoV-2 RNA PCR positive or negative laboratory result in Ontario, Canada	--	Test Request (TR)/LOINC codes for SARS-CoV-2 and other respiratory virus testing: TR12936-1, TR12937-9, 94315-9, 94314-2, 94316-7, XON13512-9, XON13529-3, XON13528-5, XON13531-9, XON13527-7. These codes, plus keywords such as “COVID”, “SARS-CoV-2”, “Novel coronavirus” or “nCOV” or microorganism SNOMED codes (840533007 [SARS-CoV-2], 168209000 [No Virus Identified]), were used to define the data pull from OLIS.	For the ICES methodology and Python script for cleaning and parsing OLIS lab results for SARS-CoV-2 and other respiratory viruses, see https://github.com/icescentral/COVID19-Lab-Results
	SARS-CoV-2 specimen date	Sex or birth date is missing or inconsistent in OLIS and RPDB	--	OLIS, and Registered Persons Database (RPDB) - contains demographic information and encrypted healthcare numbers for all OHIP eligible individuals	--
	Same as above	Death occurred > 1 day before the specimen date	Discharge disposition is not alive (DAD, NACRS)	RPDB	--
	Same as above	Non-Ontario resident	--	OLIS, RPDB	--
Study exposures	January 2007 to December 2019	ABO blood group	--	LOINC codes 882-1, 883-9, 10331-7 in OLIS	--

	SARS-CoV-2 specimen date January 15, 2020 to June 30, 2020	SARS-CoV-2 positive test (if > 1 test per person, the first positive test was used; otherwise the first negative test was used).	--	OLIS	--
Main study outcome of pulmonary embolism or deep vein thrombosis	ABO specimen date (the first time zero used in the study) to July 31, 2020.<br><br>or<br><br>SARS-CoV-2 specimen date (the second time zero used in the study) to July 31, 2020. Censored at death.	Pulmonary embolism	ICD-10-CA (DAD, SDS, NACRS): I260, I269, O88201, O88202, O88203, O88204, O88209<br><br>AND<br><br>one of the following CCI codes for diagnostic imaging during the same admission:<br>3KX30DA, 3KX30DB, 3KX30DC, 3KX30DD, 3KR10VC, 3KR10VN, 3KR12VA, 3KX10VA, 3KX10VC, 3KX10VN, 3KX10VX, 3KX12VA, 3IM10VC, 3IM10VX, 3IM10VY, 3IM12VA, 3GT70CA, 3GT70CC, 3GT70CE, 3GT70KC, 3GT70KD, 3GT70KE, 3JY10VA, 3JY10VC, 3JY10VN, 3JY10VX, 3JY12VA , 3JY20WC	OHIP ICD-9: 677, 415<br><br>AND<br><br>one of the following OHIP radiological professional fee codes for a VTE diagnostic test billed within 3 days: J198, J498, J193, J493, J202, J502, J206, J506, J182, J482, X406, X407, X125, X188, X401, X405, X408, X126, X410, X231, X232, X233, X127, X413, X421, X425, J659, J660, J859, J860	--

	Same as above	Deep vein thrombosis	ICD-10-CA (DAD, SDS, NACRS): I636, I822, I828, I829, I801, I802, I803, I808, I809, O22301, O22303, O22309, O22501, O22503, O22509, O87102, O871.04, O87109, O87304, O87309<br><br><u>AND</u><br><br>one of the following CCI codes for diagnostic imaging during the same admission: 3KX30DA, 3KX30DB, 3KX30DC, 3KX30DD, 3KR10VC, 3KR10VN, 3KR12VA, 3KX10VA, 3KX10VC, 3KX10VN, 3KX10VX, 3KX12VA, 3IM10VC, 3IM10VX, 3IM10VY, 3IM12VA, 3GT70CA, 3GT70CC, 3GT70CE, 3GT70KC, 3GT70KD, 3GT70KE, 3JY10VA, 3JY10VC, 3JY10VN, 3JY10VX, 3JY12VA , 3JY20WC	OHIP ICD-9: 671, 451<br><br><u>AND</u><br><br>one of the following OHIP radiological professional fee codes for a VTE diagnostic test billed within 3 days: J198, J498, J193, J493, J202, J502, J206, J506, J182, J482, X406, X407, X125, X188, X401, X405, X408, X126, X410, X231, X232, X233, X127, X413, X421, X425, J659, J660, J859, J860	--
Covariates	ABO specimen date onward (the first time zero used in the study)<br><br>or<br><br>SARS-CoV-2 specimen date onward (the second time zero used in the study)	Age	--	RPDB	--
	Same as above	Sex	--	RPDB	--

	Within 5 years before the ABO specimen date (the first time zero used in the study)<br><br>or<br><br>Within 5 years before the SARS-CoV-2 specimen date (the second time zero used in the study)	History of pulmonary embolism, deep vein thrombosis, or other venous thromboembolism	ICD-10-CA (DAD, SDS, NACRS): I260, I269, O88201, O88202, O88203, O88204, O88209, I636, I822, I828, I829, I801, I802, I803, I808, I809, O22301, O22303, O22309, O22501, O22503, O22509, O87102, O87104, O87109, O87304, O87309, I676, I81, I820, I823, O228, O229, O878, O879<br><br><u>AND</u><br><br>one of the following CCI codes for diagnostic imaging during the same admission: 3KX30DA, 3KX30DB, 3KX30DC, 3KX30DD, 3KR10VC, 3KR10VN, 3KR12VA, 3KX10VA, 3KX10VC, 3KX10VN, 3KX10VX, 3KX12VA, 3IM10VC, 3IM10VX, 3IM10VY, 3IM12VA, 3GT70CA, 3GT70CC, 3GT70CE, 3GT70KC, 3GT70KD, 3GT70KE, 3JY10VA, 3JY10VC, 3JY10VN, 3JY10VX, 3JY12VA , 3JY20WC	OHIP ICD-9: 677, 415, 671, 451, 452<br><br><u>AND</u><br><br>one of the following OHIP radiological professional fee codes for a VTE diagnostic test billed within 3 days: J198, J498, J193, J493, J202, J502, J206, J506, J182, J482, X406, X407, X125, X188, X401, X405, X408, X126, X410, X231, X232, X233, X127, X413, X421, X425, J659, J660, J859, J860	--
	Same as above	History of malignancy	ICD-10-CA (DAD, SDS, NACRS): C00-C97	--	--
	Same as above	Chronic kidney disease (CKD)	<u>CKD diagnosis</u><br>ICD-10-CA (DAD, NACRS): E102, E112, E132, E142, I12, I13, N08, N18, N19	<u>CKD diagnosis</u><br>ICD-9 (OHIP): 403, 585	https://pubmed.ncbi.nlm.nih.gov/23560464/

			Chronic dialysis<br>At least 2 of the following CCI (DAD, SDS) codes separated by 90 days, but < 150 days, in the year before the index date: 1PZ21	Chronic dialysis<br>At least 2 of the following OHIP fee codes separated by 90 days, but < 150 days, in the year before the index date: R849, G082, G083, G085, G090-G096, G294, G295, G323, G325, G326, G330-G333, G860-G866, H540, H740<br><br>Treatment codes (CORR): 060, 111, 112, 113, 121, 122, 123, 131, 132, 133, 141, 151, 152, 211, 221, 231, 241, 242, 251, 252, 311, 312, 313, 321, 322, 323, 331, 332, 333, 413, 423, 433, 443, 453	https://pubmed.ncbi.nlm.nih.gov/20613656/
			Exclude kidney transplant<br>CCI (DAD): 1PC85	Exclude kidney transplant<br>OHIP fee codes: S435, S434<br><br>CORR treatment code: 171 plus ≥ 1 Transplanted Organ Code [1-3]: 10, 11, 12, 18, 19	https://pubmed.ncbi.nlm.nih.gov/26019887/

	Same as above	History of cardiac ischemia	At least 1 hospitalization (DAD) or ED (NACRS) visit with a diagnosis or procedure coded with 1 of the following codes:<br><br>Angina:<br>ICD-10-CA: I20, I2382, I24<br><br>Chronic Ischemic Heart Disease:<br>ICD-10-CA: I25<br><br>Myocardial infarction:<br>ICD-10-CA: I21, I22<br><br>Coronary Artery Bypass Grafting:<br>CCI: 1IJ76, 1IJ80<br><br>Percutaneous Coronary Intervention:<br>CCI: 1IJ26, 1IJ50, 1IJ55, 1IJ57	--	https://pubmed.ncbi.nlm.nih.gov/20847972/

	Same as above	History of cardiac arrhythmia	At least 1 hospitalization (DAD) or ED (NACRS) visit with a diagnosis or procedure coded with 1 of the following codes:<br><br><u>Atrial Fibrillation/Atrial Flutter:</u><br>ICD-10-CA: I48<br><br><u>Ventricular Arrhythmia & Tachycardia:</u><br>ICD-10-CA: I470, I472, I490, I493<br><br><u>Permanent Pacemaker:</u><br>CCI: 1HZ53GRNM, 1HZ53LANM, 1HZ53GRNK, 1HZ53LANK, 1HZ53GRNL, 1HZ53LANL<br><br><u>Implantable Cardioverter- Defibrillator:</u><br>CCI: 1HZ53GRFS, 1HZ53LAFS, 1HZ53SYFS, 1HZ53HAFS	--	https://pubmed.ncbi.nlm.nih.gov/19433698/<br><br>https://www.ices.on.ca/Publications/Atlases-and-Reports/2006/Canadian-Institute-for-Health-Information<br><br>https://pubmed.ncbi.nlm.nih.gov/17599603/
	Any time before the ABO specimen date (the first time zero used in the study)<br><br>or<br><br>Any time before the SARS-CoV-2 specimen date (the second time zero used in the study)	History of heart failure	The ICES-derived CHF database was used to identify patients with CHF, based on 1 ED, hospitalization or outpatient claim, and a second claim in 1 year. *The CHF database is limited to those 40 years of age or older.*<br><br>ICD-10-CA (DAD, SDS): I500, I501, I509	OHIP ICD-9: 428	https://pubmed.ncbi.nlm.nih.gov/23735455/

	Same as above	Diabetes mellitus	The ICES-derived ODD database was used to identify patients with diagnosed diabetes before the index date, based on 2 OHIP diagnostic codes or 1 OHIP fee code or 1 DAD/SDS diagnostic code, within 2 years.<br><br>ICD-10-CA: E10, E11, E13, E14	OHIP ICD-9: 250<br>OHIP fee codes: Q040, K029, K030, K045, K046	https://pubmed.ncbi.nlm.nih.gov/11874939/
Other baseline variables	SARS-CoV-2 specimen date	Area income quintile	--	Statistics Canada Census	--
	Any time before the SARS-CoV-2 specimen date	Asthma	The ICES-derived ASTHMA database was used to identify patients with diagnosed asthma before the index date, based on 2 OHIP diagnostic codes or 1 DAD diagnostic code.<br><br>ICD-10-CA: J45, J46	OHIP ICD-9: 493	https://pubmed.ncbi.nlm.nih.gov/20011725/
	Same as above	Chronic obstructive pulmonary disease (COPD)	The ICES-derived COPD database was used to identify patients with diagnosed COPD before the index date, based on 1 OHIP diagnostic code or 1 DAD diagnostic code.<br><br>ICD-10-CA: J41-J44	OHIP ICD-9: 491, 492, 496	https://pubmed.ncbi.nlm.nih.gov/19863368/ *The COPD algorithm was validated in those aged ≥ 35 years.*
	Same as above	Chronic hypertension	The ICES-derived HYPER database was used to identify patients with:<br>a) 1 hospital admission with a hypertension diagnosis, or<br>b) an OHIP claim with a hypertension diagnosis followed within 2 years by either an OHIP claim or a hospital admission with a hypertension diagnosis.<br><br>ICD-10-CA (DAD, SDS): I10-I13, I15	OHIP ICD-9: 401-405	https://pubmed.ncbi.nlm.nih.gov/20101286/

	Same as above	Immunocompromised (HIV or organ transplant)	--	The ICES-derived HIV database was used to identify patients with pre-existing HIV, based on 3 physician claims in 3 years.<br><br>OHIP ICD-9: 042-044<br><br>CORRLINK links CORR and DAD data and includes patients who received an organ transplant, and does not include dialysis patients.	https://pubmed.ncbi.nlm.nih.gov/21738786/
	Same as above	Dementia	The ICES-derived DEMENTIA database was used to identify individuals with 1 hospitalization for dementia and/or 3 outpatient visits for dementia, each separated by 30 days, within 2 years, or 1 prescription from ODB.<br><br>ICD-10-CA (DAD, SDS): F00-F03, G30	OHIP ICD-9: 290, 331<br><br>ODB<br>1 prescription for a cholinesterase inhibitor	https://pubmed.ncbi.nlm.nih.gov/27567819/
	Within 1 year before the SARS-CoV-2 specimen date	Frailty	Identified based on the following rules, using DAD and OHIP databases:<br>1. Long-term care residence (i.e., admitted from/discharged to, a nursing home after hospital stay, or location of physician billing claim was long-term care facility);<br>2. Receipt of palliative care;<br>3. Two or more domains derived from frailty scales (i.e., cognitive impairment, falls, general health status, incontinence, nutrition issues, functional performance) and health services utilization (i.e., ≥ 2 hospital stays or ED visits, geriatrician or home care visit).	--	https://pubmed.ncbi.nlm.nih.gov/28974280/

	Within 5 years before the SARS-CoV-2 specimen date	Anemia	ICD-10-CA (DAD, SDS, NACRS): D50-D53, D55, D56, D572-D574, D58-D61, D63, P55, P560, P570	OHIP ICD-9: 280-285, 773	--
	Same as above	Transient ischemic attack or acute ischemic stroke	Transient Ischemic Attack:<br>At least 1 hospitalization or ED visit with 1 of the following diagnosis codes:<br><br>ICD-10-CA (DAD, NACRS): G450-G453, G458, G459, H340<br><br>Acute Ischemic Stroke:<br>1 hospitalization with a main diagnosis coded with one of the following codes:<br><br>ICD-10-CA (DAD): I63 (except I636), I64, H341	--	http://canadianstrokenetwork.ca/en/wp-content/uploads/2014/08/Stroke_Core_ENG.pdf

ASTHMA: Ontario Asthma dataset; CCI: Canadian Classification of Interventions; CHF: Ontario Congestive Heart Failure dataset; CIHI: Canadian Institute for Health Information; CORR: Canadian Organ Replacement Registry; DAD: Discharge Abstract Database; DEMENTIA: Ontario Dementia dataset; ED: Emergency Department; HIV: Ontario HIV dataset; HYPER: Ontario Hypertension dataset; ICD-9: International Classification of Diseases, 9th Revision; ICD-10-CA: International Classification of Diseases, 10th Revision, Canada; ICU: Intensive Care Unit; LHIN: Local Health Integration Network; LOINC: Logical Observation Identifiers Names and Codes; NACRS: National Ambulatory Care Reporting System; ODB: Ontario Drug Benefit; ODD: Ontario Diabetes Dataset; OHIP: Ontario Health Insurance Plan; OLIS: Ontario Laboratories Information System; RPDB: Registered Persons Database; SDS: Same Day Surgery
